# Omega-3 intake is associated with attenuated inflammatory response and cardiac remodeling after myocardial infarction

**DOI:** 10.1186/s12937-019-0455-1

**Published:** 2019-05-06

**Authors:** Alessandra M. Campos-Staffico, Ana Paula R. Costa, Luiz Sérgio F. Carvalho, Filipe A. Moura, Simone N. Santos, Otavio R. Coelho-Filho, Wilson Nadruz, José C. Quinaglia e Silva, Andrei C. Sposito, on behalf of Brasilia Heart Study

**Affiliations:** 1 Cardiology Department, State University of Campinas (Unicamp), Campinas, SP Brazil; 2 Medical School, University of Brasilia (UnB), Brasília, DF Brazil; 3 Department of Medicine, Weill-Cornell Medical College, New York, United States; 4 Hospital de Base do Distrito Federal, Brasília, Brazil

**Keywords:** Omega-3, Inflammatory response, Cardiac remodeling, STEMI

## Abstract

**Background:**

Myocardial infarction (MI) elicits an intense acute inflammatory response that is essential for cardiac repair. However, an excessive inflammatory response also favors myocardial apoptosis, cardiac remodeling, and cardiovascular mortality. Omega-3 polyunsaturated fatty acids (ω-3) bear anti-inflammatory effects, which may mitigate the inflammatory response during MI. This study investigated whether ω-3 intake is associated with attenuation of the MI-related inflammatory response and cardiac remodeling.

**Methods:**

ST-elevation MI (STEMI) patients (*n* = 421) underwent clinical, biochemical, nutritional, 3D echocardiogram, Cardiac Magnetic Resonance imaging (CMRi) at 30 days and 3D echocardiogram imaging at six months after the MI. Blood tests were performed at day one (D1) and day five (D5) of hospitalization. Changes in inflammatory markers (ΔD5-D1) were calculated. A validated food frequency questionnaire estimated the nutritional consumption and ω-3 intake in the last 3 months before admission.

**Results:**

The intake of ω-3 below the median (< 1.7 g/day) was associated with a short-term increase in hs-C-reactive protein [OR:1.96(1.24–3.10); *p* = 0.004], Interleukin-2 [OR:2.46(1.20–5.04); *p* = 0.014], brain-type natriuretic peptide [OR:2.66(1.30–5.44); *p* = 0.007], left-ventricle end-diastolic volume [OR:5.12(1.11–23.52)]; *p* = 0.036] and decreases in left-ventricle ejection fraction [OR:2.86(1.47–6.88); *p* = 0.017] after adjustment for covariates. No differences were observed in the extension of infarcted mass obtained by CMRi.

**Conclusion:**

These findings suggest that a reduced daily intake of ω-3 may intensify outcome-determining mechanisms after STEMI, such as acute inflammatory response and late left ventricular remodeling.

**Trial registration:**

Clinical Trial Registry number and website: NCT02062554.

## Introduction

Myocardial infarction (MI) elicits an intense acute inflammatory response due to mechanical stress and ischemia-reperfusion injury [1]. The generation of pro-inflammatory cytokines via activation of both Nuclear Factor κB (NF-κB) and Activator Protein-1 (AP-1) transcription factors represents a central step in the triggering of this response [2–4]. Although this phenomenon is involved for cardiac repair, excessive inflammation favors myocardial apoptosis and cardiac remodeling, thus leading to increased cardiovascular mortality [5–7].

Omega-3 (ω-3) are polyunsaturated fatty acids whose interactions with human physiology have been reported, particularly for α-linolenic acid (ALA), eicosapentaenoic acid (EPA) and docosahexaenoic acid (DHA). EPA and DHA components bear anti-inflammatory effects through two complementary actions: (i) reducing the content of arachidonic acid products and (ii) downregulating the expression of pro-inflammatory cytokines via NF-κB and AP-1 inhibition [8–12]. The clinical impact of this set of mechanisms has been documented in several conditions involving the acute phase response. These included sepsis and chronic inflammatory conditions such as rheumatoid arthritis, multiple sclerosis, type 1 diabetes mellitus, end-stage renal disease, and Alzheimer disease [13–19]. Considering the abovementioned premises, we hypothesized that MI-related inflammatory response would be attenuated in individuals who had enhanced ω-3 intake prior the onset of the coronary event. This assumption is consistent with a previous randomized controlled trial that showed a decrease in cardiovascular death with dietary supplementation with ω-3 [20]. However, it is unclear whether the acute inflammatory response and cardiac remodeling may be further subsided by ω-3 intake that is already established at the time of the inciting event. Therefore, aiming to investigate whether ω-3 intake is associated with attenuation of the MI-related inflammatory response and cardiac remodeling, we analyzed a prospective cohort database originally designed to include the investigation of nutritional characteristics from 3 months prior to 3 months after ST-elevation MI (STEMI) manifestation.

## Subjects and methods

### Study design and participants

**Fig. 1:**
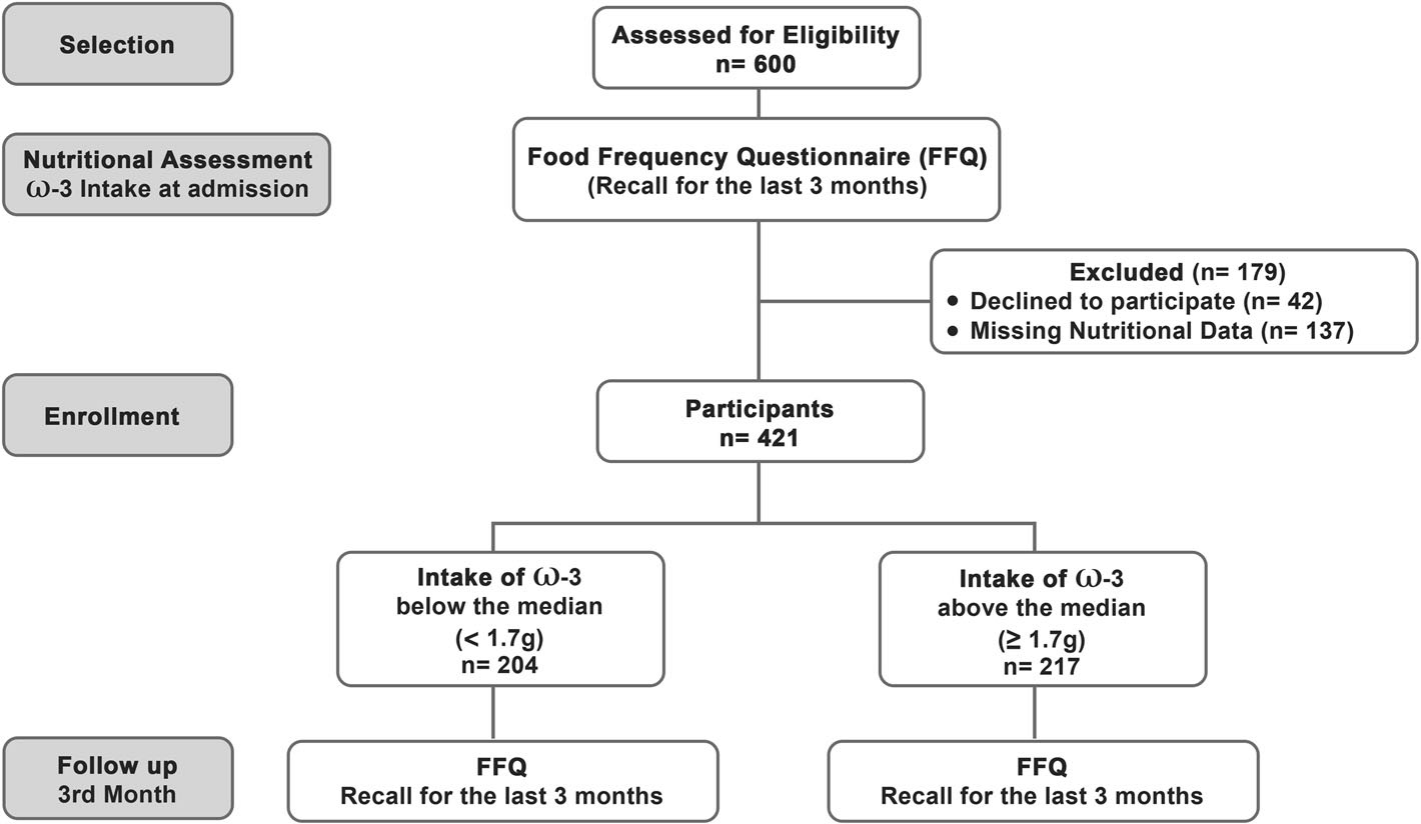
Diagram flow of study design

Participants from the Brasília Heart Study (BHS) database were used for this analysis (*n* = 421). BHS is a an ongoing prospective observational cohort composed of STEMI patients admitted to Hospital de Base do Distrito Federal (Brasília, Brazil) since 2006 [21]. Briefly, BHS inclusion criteria were: (i) < 24 h after onset of MI symptoms, (ii) ST-segment elevation of at least 1-mm (frontal plane) or 2-mm (horizontal plane) in 2 contiguous leads and (iii) increased myocardial necrosis markers above reference limit of creatine kinase-MB (CK-MB) (25 U/L) and troponin I (0.04 ng/mL) followed by a decline of both markers. Participants with new or presumed left bundle branch block, development of pathological Q waves without ST-elevation recording or exclusively with imaging evidence of MI were excluded. Diagram flow is showed in Fig. 1.

Patients enrolled into the study underwent therapies based on current guidelines for treatment of acute coronary disease, as indicated by Table 1 data [22]. Attending physicians, who were blind to the study evaluations, made all therapeutic decisions.

**Table 1:** Baseline characteristics of clinical and laboratory data of participants

Characteristics	< 1.7 g *n* = 204	≥1.7 g *n* = 217	*p*-value
Daily intake of ω-3 at admission (g)	1.0(0.7)	2.7(1.3)	< 0.001
Daily intake of ω-3 at 3 months (g)	1.1(0.9)	1.9(1.5)	< 0.001
Age, (years)	60 ± 10	58 ± 11	0.256
Gender: female n(%)	52(26)	52(24)	0.717
Diabetes mellitus, n(%)	31(15)	55(25)	0.010
Hypertension, n(%)	113(56)	141(65)	0.044
Previous myocardial infarction, *n*(%)	21(10)	22(10)	0.957
Previous stroke, *n*(%)	11(5)	11(5)	0.881
Coronary reperfusion therapy, *n*(%)	171(84)	194(89)	0.012
Percutaneous coronary intervention (PCI), *n*(%)	94(46)	100(46)	0.479
Fibrinolytic therapy, *n*(%)	125(61)	151(70)	0.052
PCI and fibrinolytic therapy, *n*(%)	53(26)	65(30)	0.190
Current smoking, *n*(%)	78(38)	83(39)	0.970
Ex smoking, *n*(%)	59(29)	70(33)	0.440
Drugs previously in use:			
Statin, *n*(%)	13(6)	13(6)	0.861
Calcium channel blocker, *n*(%)	20(10)	22(10)	0.922
Beta-blocker, *n*(%)	40(20)	32(14)	0.139
Angiotensin inhibitor drugs (ACEi or ARB), *n*(%)	66(32)	93(43)	0.026
Aspirin, *n*(%)	34(17)	33(15)	0.666
HbA1c, (%)	6.0(0.8)	6.1(1.2)	0.830
Glomerular filtration rate, (mL/min)	70(24)	69(25)	0.173
Triglycerides, (mg/dL)	118(90)	147(126)	0.001
LDL-C, (mg/dL)	118 ± 38	121 ± 48	0.538
HDL-C, (mg/dL)	38(13)	36(13)	0.150
Systolic blood pressure, (mmHg)	130(37)	140(40)	0.016
Diastolic blood pressure, (mmHg)	80(30)	85(26)	0.063
Heart rate, (bpm)	75(18)	75(25)	0.415
Time between MI onset and medical care, (minutes)	120(255)	90(182)	0.126
Killip >I, *n*(%)	20(10)	26(12)	0.470
Peak CK-MB, (UI/L)	161(243)	200(268)	0.070
Troponin, (ng/mL)	2.40(22.36)	1.52(20.16)	0.468
Body Mass Index, (kg/m^2^)	25.9(5.1)	26.8(5.0)	0.234
Waist circumference, (cm)			
Female	95(14)	93(9)	0.164
Male	95(13)	98(12)	0.173

### Clinical evaluation

Participants underwent a structured detailed clinical questionnaire, anthropometric measurements, blood collection for biochemical analysis, nutritional, and imagining evaluation. Ex-smoking status, diabetes and hypertension were previously defined elsewhere [23]. Coronary reperfusion therapy was assumed when the participant underwent to percutaneous coronary intervention (PCI) and/or fibrinolytic therapy to restore the blood flow during MI. Anthropometrical measurements obtained were body weight (kg), height (m), and waist circumference (WC). Killip and GRACE scores were evaluated in all enrolled patients. Briefly, the GRACE risk score is a well-validated multivariable algorithm for predicting 10-year death risk following an initial acute coronary syndrome (ACS) [24].

### Biochemical analysis

Blood samples were obtained within the first 24 h of MI symptoms (D1) and at day five of hospitalization (D5). High-sensitivity C-reactive protein (hs-CRP), urea, creatinine, triglycerides, total cholesterol, high-density lipoprotein cholesterol (HDL-C), low-density lipoprotein cholesterol (LDL-C), and glomerular filtration rate (GFR) were obtained as described elsewhere [23]. Interleukin-2 and B-type natriuretic peptide levels were quantified by ELISA.

### Nutritional evaluation

A food frequency intake questionnaire (FFQ) was used to estimate nutritional composition and ω-3 intake [alpha-linolenic acid (ALA), eicosapentaenoic acid (EPA), and docosahexaenoic acid (DHA)] within the previous 3-month period. The questionnaire was applied at admission and at the 3rd month of follow-up. The food intake was clustered in 62 items divided into 10 groups (dairy products, eggs and meats, oils, snacks and canned foods, cereals and vegetables, greens and fruits, desserts and candies, beverages, diet and light products and spices). Approximate portions of usual intake of each item was recalled by patients with the aid of a photographic record for dietary surveys and the portion size was, subsequently, quantified into weights [25]. Total caloric and ω-3 intake were calculated based on a food composition database of the Brazilian Table of Food Composition (TACO) [26]. Briefly, TACO is a 399-item nutritional database with the most consumed foods in Brazil. Sample of foods were collected in 9 cities in all 5 Brazilian regions. Sampling was performed by mixing and packing two units of each principal brand collected in each site. The total units were separated in three different composites of 100–200 g, and then analyzed by certified laboratories. Contents of total lipids, cholesterol, saturated, poly- and monounsaturated fatty acids and trans fats were entered in the database [27]. Quantification of ω-3 from the questionnaires was performed by an experienced nutritionist (A.P.R.C.) and values were omitted from all other study participants.

### Echocardiography

Participants underwent 3D Echocardiography at the first- and sixth-month following MI (iE 33 system; Philips Medical Systems, Andover, MA) according to current guidelines [28]. Left ventricle end-diastolic diameter (LVEDD), end-systolic diameter (LVESD), septum diastolic thickness (SD), and posterior wall diastolic thickness (PD), left ventricle end-diastolic volume (LVEDV) and end-systolic volume (LVESV) were obtained in 3D mode, and Ejection fraction (EF) was derived from these volumes.

### Cardiac magnetic resonance imagining

Cardiac Magnetic Resonance imaging (CMRi) and CMRi data analysis were performed according to descriptions shown in a previous study [29].

### Statistical analysis

Normally distributed data were presented as mean ± SD and skewed data as median and interquartile range (IQR). Participants were stratified into two groups according to daily consumption of ω-3 (below or above the median). Chi-square or two-tailed *t* tests were used for comparison of baseline data. Analyses of covariance (ANCOVA) was used to assess the association between ω-3 consumption and the change (Δ) of hs-CRP, IL-2 and BNP levels between D1 and D5. ANCOVA adjusted by covariates was also used to compare infarcted mass and the change in LVEF and LVEDV between 30 and 180 days. All analyses of the changes between D1 and D5 or between the 30th and the 180th day were additionally adjusted for the baseline levels in order to attenuate the effect of regression toward the mean. Multivariate binary logistic regression was used to evaluate the association between the dichotomous dependent variable ω-3 intake below or above the median and the independent variables Δhs-CPR, ΔIL-2, ΔBNP, ΔLVEF and ΔLVEDV. These dependent variables were categorized into below or above their respective medians to bypass the non-normal distribution and as a strategy to level their effects sizes, allowing direct comparability between variables. Stepwise selection of variables was used to reach the final model using the covariates. The following covariates were used for ANCOVA and regression analyses and were selected using bootstrapping based on *t* test analysis (Table 1): age, gender, diabetes mellitus, hypertension, coronary reperfusion therapy, and use of ACE inhibitors/ARBs. Restricted cubic spline models were used to assess the relationship between daily consumption of ω-3 and hs-CRP levels. Splines were adjusted by the GRACE score [24] and plasma peak CKMB levels. The GRACE score was chosen because its validity was confirmed as a predictive instrument in STEMI patients and due to the possibility of aggregating into a single index relevant covariate, thus reducing the saturation of the regression models. Statistical analysis was performed using SPSS®, version 21 for Mac (IBM) and STATA, version 15.0 for Mac. A two-sided *p*-value of 0.05 was considered statistically significant.

## Results

Baseline characteristics are shown in Table 1. Participants who had a daily intake of ω-3 above the median (≥1.7 g/day) at admission had higher systolic blood pressure, higher levels of triglycerides and higher frequency of diabetes mellitus, hypertension, and coronary reperfusion therapy as well as the use of ACE inhibitors/ARBs than their counterparts.

### Nutritional follow-up

Three months after STEMI, the food questionnaire was reapplied in order to evaluate the intake of ω-3 in this interlude. Participants who originally presented with ω-3 intake below the median (< 1.7 g/day) had no changes in their ω-3 intake during follow-up (from 1.0(0.7) to 1.1(0.9) g/day; *p* = 0.572). However, participants who originally had an intake of ω-3 above the median (≥1.7 g/day) reduced their ω-3 intake after STEMI (from 2.7(1.3) to 1.9(1.5) g/day; *p* < 0.001). Due to the natural limitation of the FFQ tool, it is not possible to specify the difference of 30% (0.8 g). Regardless, after 3 months, the higher intake group were still consuming significantly higher amounts of ω-3 (Table 1).

### Inflammatory markers

Comparative analyses of inflammatory markers are shown in Table 2. Participants who consumed ω-3 above or below the median had equivalent levels of hs-CRP at D1. Participants who consumed ω-3 above the median had higher levels of IL-2 at D1 than their counterparts. The increase in both hs-CRP and IL-2 levels from D1 to D5 were smaller among those who consumed ω-3 above the median than their counterparts. All these comparative results remained significant after adjustment for covariates as indicated above.

**Table 2:** Comparative levels of inflammatory markers, BNP levels, LVEF and infarcted mass

Characteristics	< 1.7 g *n* = 204	≥1.7 g *n* = 217	*p*-value	ANCOVA^a^
Inflammatory markers				
hs-CRP at 1st day, (mg/L)	0.60(1.11)	0.60(0.97)	0.820	0.648
hs-CRP at 5th day, (mg/L)	4.10(6.90)	2.90(4.62)	0.005	0.027
Δhs-CRP, (mg/L)^b^	+ 2.80(5.50)	+ 1.57(4.14)	0.001	0.004
IL-2 at 1st day, (pg/mL)	0.04(1.24)	1.34(2.60)	< 0.001	0.036
IL-2 at 5th day, (pg/mL)	5.83(6.55)	5.46(6.30)	0.110	0.224
**Δ**IL-2, (pg/mL)^b^	+5.36(6.18)	+ 3.87(6.17)	0.003	0.013
Cardiac remodeling marker				
BNP at 1st day, (ng/mL)	0.18(0.13)	0.17(0.17)	0.208	0.099
BNP at 5th day, (ng/mL)	0.35(0.32)	0.21(0.26)	< 0.001	0.056
ΔBNP, (ng/mL)^b^	+ 0.14(0.27)	+ 0.07(0.18)	0.003	0.029
Cardiac Magnetic Resonance Imaging and Echocardiography				
Infarcted mass, (%)	12.6 ± 7.3	11.8 ± 7.1	0.311	0.406
Infarcted mass, (g)	15.6(9.3)	14.6(11.9)	0.628	0.798
LVEF at 1st month, (%)	55.0 ± 14.8	53.7 ± 13.8	0.590	0.900
LVEF at 6th month, (%)	54.7 ± 12.8	59.9 ± 10.6	0.189	0.609
ΔLVEF, (%)^b^	−1.9 ± 10.7	+ 4.5 ± 13.6	0.165	0.010
LVEDV at 1st month, (mL)	89.8 ± 38.0	96.6 ± 32.6	0.580	0.960
LVEDV at 6th month, (mL)	99.2 ± 43.5	85.6 ± 22.9	0.223	0.848
ΔLVEDV, (%)^b^	+ 13.2 ± 22.8	−8.8 ± 14.7	0.001	0.011

^a^Adjusted for diabetes mellitus, hypertension, coronary reperfusion therapy and use of ACEi/ARBs drugs

^b^All delta (Δ) variables were also adjusted for their baseline level at D1

Binary logistic regression models are shown in Table 3 and were used to assess the association between the intake of ω-3 and study endpoints. The intake of ω-3 was inversely associated with increase in hs-CRP and IL-2 levels (Δhs-CRP and ΔIL-2 – Model 1). Both associations remained significant after full adjustment for covariates (Model 2). The addition of smoking habit as covariate did not change the results.

**Table 3:** Binary logistic regression to assess the association between daily consumption of omega-3 and Δhs-CRP, ΔIL-2, ΔBNP, ΔLVEF and ΔLVEDV

	OR(95%CI); *p*-value Δhs-CRP ≥ + 2.20 mg/L
Omega-3 < 1.7 g	
Model 1	1.819(1.226–2.699);0.003
Model 2	1.958(1.238–3.097);0.004
	OR(95%CI); *p*-value ΔIL-2 ≥ + 4.642 pg/mL
Omega-3 < 1.7 g	
Model 1	2.092(1.123–3.897);0.020
Model 2	2.464(1.205–5.038);0.014
	OR(95%CI); *p*-value ΔBNP ≥ + 0.10 ng/mL
Omega-3 < 1.7 g	
Model 1	2.481(1.300–4.737);0.006
Model 2	2.660(1.300–5.443);0.007
	OR(95%CI); *p*-value ΔLVEF < + 1.2%
Omega-3 < 1.7 g	
Model 1	4.115(1.150–14.723);0.030
Model 2	5.119(1.114–23.518);0.036
	OR(95%CI); *p*-value ΔLVEDV ≥ -3.248%
Omega-3 < 1.7 g	
Model 1	5.250(1.093–25.211);0.038
Model 2	2.857(1.467–6.877);0.017

Model 1: Unadjusted; Model 2: Adjusted for diabetes mellitus, hypertension, coronary reperfusion therapy, use of ACEi/ARBs drugs, triglycerides, systolic blood pressure, diastolic blood pressure and peak CK-MB

**Fig. 2:**
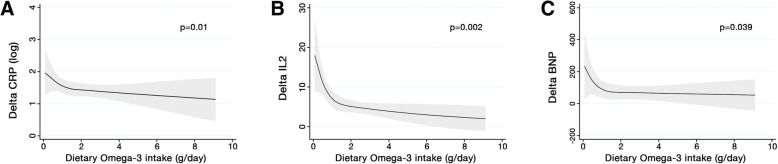
Restricted cubic spline curves to assess for relationship between daily consumption of ω-3 and change in hs-CRP (**a**), IL-2 (**b**) and BNP levels (**c**) during acute phase of STEMI (ΔCRP = D5 – D1). Splines were adjusted by the Global Registry of Acute Coronary Events (GRACE) score and plasma peak CKMB level

Restricted cubic spline models were used to assess the relation between the daily consumption of ω-3 across changes in hs-CPR levels (Fig. 2). The consumption of ω-3 was inversely associated with the change in hs-CRP levels even after fully adjusted analysis (*p* = 0.01).

### Cardiac remodeling

Comparative analyses of BNP levels, infarcted mass, LVEF, LVEDV and SI are shown in Table 2. No difference in plasma levels of BNP was observed at D1. However, BNP levels significantly increased in both subgroups from D1 to D5 (*p* < 0.001 and *p* = 0.032 for subgroups below and above the median, respectively). In the subgroup analysis, those who consumed ω-3 levels above the median had lower levels of BNP at D5 than their counterparts. Accordingly, participants who consumed ω-3 levels above the median had a smaller change in BNP levels (ΔD5-D1) than their counterparts; this difference remained significant after adjustment.

No differences were observed in the extension of infarcted mass obtained by CMRi neither in the LVEF at first and sixth months after STEMI. However, participants who consumed ω-3 levels above the median had an increase of LVEF from the first to the sixth month after STEMI while their counterparts had a decrease of LVEF in this period of time. Also, LVEDV was not different between the groups at the first and sixth month after STEMI. Nevertheless, participants who consumed ω-3 levels above the median had a decrease in LVEDV from the first to the sixth month while their counterparts had an increase in LVEDV in the same period.

The associations between the intake of ω-3 and the changes in the levels of BNP, LVEF and LVEDV measures were tested by binary logistic regression models as shown in Table 3. The intake of ω-3 below the median was inversely associated with increase in BNP levels. Conversely, the intake of ω-3 below the median was directly associated with reduction in LVEF and decrease in LVEDV. Both associations remained significant after full adjustment for covariates.

## Discussion

In short, this study indicates that an increased daily intake of ω-3 (at least 1.7 g) is associated with attenuated inflammatory response and ventricular remodeling after STEMI.

Excessive increase in pro-inflammatory cytokines secretion after MI have been associated with a higher risk for mortality [30]. Clinically, hs-CRP is a has been established as the preferred biomarker for inflammatory activity given its long half-life and its value in predicting both the incidence of cardiovascular events and mortality [31]. In STEMI patients, we previously observed a direct relationship between the activation of the innate and TH_1_ inflammatory response after post-reperfusion expansion of the peri-infarcted myocardial mass [29]. This finding has motivated us to verify the effect of such inflammatory attenuation of the left ventricle remodeling after STEMI. In fact, in line with prior studies in chronic and acute disease [13–19], individuals consuming at least 1.7 g ω-3 had a greater reduction of IL-2 (a classical marker for Th_1_ response) and hs-CRP (a marker for innate response) during the acute phase of MI.

Higher intake of ω-3 before MI was associated with higher late gain of LVEF and decreased late gain of LVEDV, indicating a lower degree of remodeling. As the nutritional reanalysis of these individuals three months after STEMI continued to point to a higher consumption of ω-3, we cannot rule out the possibility that persistently increased consumption also contributed to this outcome. We also cannot exclude the possibility of a play of chance due to the observational design of this study, precluding the balance between the groups for variables not measured or not known. Nevertheless, the treatment of high-dose of ω-3 after STEMI has been shown to be associated with a reduction in left ventricular remodeling, myocardial fibrosis, and systemic inflammation in a prospective randomized controlled trial [32]. This may result from the anti-inflammatory action of these fatty acids and may also result from direct effects of ω-3 on myocardial cells. For example, ω-3 protects the metabolic and functional properties of cardiomyocytes submitted to conditions prone to insulin resistance, such as STEMI [33]. Also, intake of certain types of fatty acids may modulate cardiomyocyte gene expression and, by this way, change myocardial bioenergetics; ω-3, for example, stimulates angiopoietin-like protein 4 resulting in decreased cardiac absorption of fatty acids and decreased oxidative stress induced by fatty acids and lipid peroxidation [34]. Thus, the toning down of both systemic inflammatory activation and ventricular remodeling after STEMI may result from a combination of direct and indirect effects of ω-3.

Some limitations must be considered when reading this study. Firstly, as commented above, our findings must be considered as hypothesis generating due to the observational design and hence the impossibility to exclude selection bias. Randomized controlled trials (RCT) are required to exclude unbalance between the arms and the healthy cohort effect. However, consistency with RCT and mechanistic data deem this finding plausible. Other limitation that should be considered is the recall bias due to FFQ application, since participants are asked to report their food intake retrospectively in a prolonged period of time.

## Conclusion

In conclusion, the findings of the present study suggest that a reduced daily intake of ω-3 may intensify outcome-determining mechanisms after STEMI, such as acute inflammatory response and late left ventricular remodeling.

## Abbreviations

ACEi: Angiotensin-converting enzyme inhibitors
ANCOVA: Analysis of covariance
AP-1: Activator Protein-1
ARB: Angiotensin II receptor blockers
BHS: Brasília Heart Study
BNP: B-type natriuretic peptide
CK-MB: Creatine kinase myocardial bound
CMRi: Cardiac Magnetic Resonance imaging
D1: First day of hospitalization
D5: Fifth day of hospitalization
FFQ: Food frequency questionnaire
hs-CRP: High-sensitivity C-reactive protein
IL-2: Inteleucin-2
LVEDD: Left ventricle end-diastolic diameter
LVEDV: Left ventricle end-diastolic volume
LVEF: Left ventricle ejection fraction
LVESD: End-systolic diameter
LVESV: Left ventricle end-systolic volume
MI: Myocardial infarction
NF-κB: Nuclear Factor κB
PD: Posterior wall diastolic thickness
SD: Septum diastolic thickness
STEMI: ST-elevation myocardial infarction
ΔD5-D1: Change from fifth to first day of hospitalization
ω-3: Omega-3
